# Tumor cells containing the African-Centric S47 variant of *TP53* show increased Warburg metabolism

**DOI:** 10.18632/oncotarget.26660

**Published:** 2019-02-05

**Authors:** Thibaut Barnoud, Joshua L.D. Parris, Maureen E. Murphy

**Affiliations:** ^1^ Program in Molecular and Cellular Oncogenesis, The Wistar Institute, Philadelphia PA 19104, USA; ^2^ Cell and Molecular Biology Program, Perelman School of Medicine at the University of Pennsylvania, Philadelphia PA 19104, USA

**Keywords:** p53, Pro47Ser, metabolism, RRAD, mitochondria

## Abstract

Mutations in the *TP53* tumor suppressor gene remain a hallmark of human cancer. In addition to mutation of *TP53*, single nucleotide polymorphisms (SNPs) in this gene can have a profound impact on p53 function, and can affect cancer risk as well as other p53 functions. Wild type (WT) p53 contains a proline at amino acid 47, but approximately 1% of African-Americans express a p53 allele with a serine at amino acid 47 (Pro47Ser, hereafter S47). In a mouse model for this variant, mice expressing S47 are predisposed to spontaneous cancers. The S47 variant also is associated with increased pre-menopausal breast cancer risk in African American women. We recently reported that S47 tumor cells are resistant to the majority of cytotoxic chemotherapeutic agents, but show increased sensitivity to a subset of anti-cancer agents, compared to tumors with WT p53. In this work, we report on another potentially promising therapeutic vulnerability of S47 tumors. We find that S47 tumors show decreased mitochondrial metabolism, along with increased dependency on glycolysis. S47 tumor cells also show increased sensitivity to the glycolytic poison 2-deoxy-glucose. We propose that the altered metabolism in S47 tumor cells may be yet another potentially-actionable therapeutic vulnerability to exploit in cancer-prone individuals with this genotype.

## INTRODUCTION

The *TP53* tumor suppressor plays a critical role in suppressing tumorigenesis by directly regulating the expression of genes that promote apoptosis, cell cycle arrest, and senescence. In addition, p53 plays roles in ferroptosis, metabolism and autophagy, and these pathways likely contribute to the tumor suppressor functions of p53 as well [1–3]. Approximately half of all human tumors contain mutations in *TP53*. In addition to mutations, there are single nucleotide polymorphisms (SNPs) in *TP53* that dampen p53 function, and that can increase cancer risk and decrease the efficacy of cancer therapy [4]. A nonsynonymous single-nucleotide polymorphism at codon 47 in *TP53* exists in African-descent populations, and this SNP confers increased cancer risk in mice and humans [5, 6]. Approximately 6% of Africans and 1% of African-Americans express a p53 allele with a serine residue at codon 47 (Pro47Ser, rs1800371). We created a mouse model for this variant, hereafter S47, and showed that mice expressing this variant in either heterozygous or homozygous form show increased risk for hepatocellular carcinoma and other cancers [5]. We showed that the S47 variant is an intrinsically poorer tumor suppressor compared to WT p53 [7, 8]. Moreover, we identified two therapeutic agents, cisplatin and BET inhibitors, which are preferentially cytotoxic to tumor cell lines containing the S47 variant [7].

Here-in we report that S47 transformed cells also show increased glycolytic rates and decreased mitochondrial respiration, compared to tumor cells with WT p53. Our data support the premise that the increased glycolytic flux in S47 cells may provide an additional target for cancer therapy in these individuals. In support of this premise we show that S47 tumor cells are preferentially sensitive to 2-deoxy-glucose, compared to their wild type p53 counterparts. These data strengthen the argument for personalized approaches tailored to *TP53* genotype.

## RESULTS

### Tumor cells containing the S47 variant of p53 show decreased oxidative phosphorylation and increased glycolysis

**Figure 1:**
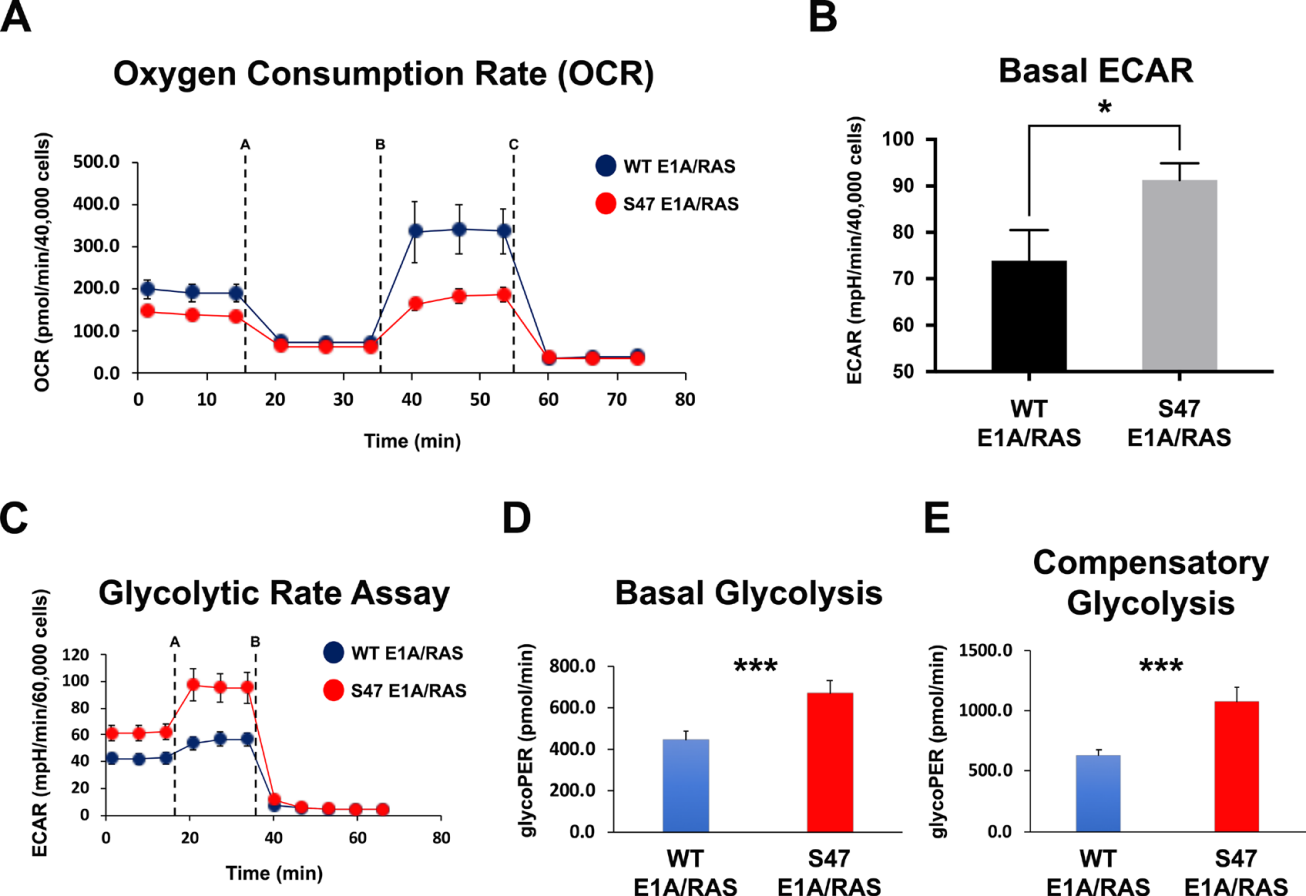
Increased use of glycolysis in tumor cells with the S47 variant of p53 (**A**) WT and S47 E1A/RAS MEFs were subjected to the Seahorse XF Cell Mito Stress Test. Each graphical representation indicates the mean ± standard deviation of technical replicates. Shown are representative data of two independent clones of each genotype. Injections were Oligomycin (1 μM, line A), FCCP (1 μM, line B), and Rotenone/Antimycin A (0.5 μM, line C). (**B**) Quantification of the basal extracellular acidification rate (ECAR) between WT and S47 E1A/RAS MEFs from the Mito Stress Test performed in (A). Each graphical representation indicates the mean ± standard deviation of technical replicates; ^*^p < 0.05. (**C**) WT and S47 E1A/RAS MEFs were subjected to the Seahorse XF Glycolytic Rate Assay. Injections were Rotenone plus Antimycin A (0.5 μM, line A), and 2-deoxy-D-glucose (2-DG, 50 mM, line B). Shown are representative data of two independent clones of each genotype. (**D**) Basal glycolysis and (**E**) compensatory glycolysis were analyzed between WT and S47 E1A/RAS MEFs. All experiments were performed in triplicate, with each group containing 5–10 technical replicates. ^***^p < 0.001. glycoPER: Glycolytic proton efflux rate.

In order to determine the mechanisms whereby the S47 variant of p53 is a poorer tumor suppressor, we previously conducted analyses of p53 target genes in cells containing WT p53 and S47 [5]. We noted that several of the p53 target genes with impaired transactivation in S47 cells are involved in metabolism. This includes SCO2 and GLS2, which are known p53 target genes involved in mitochondrial metabolism; we previously showed impaired transactivation of these genes in non-transformed S47 cells [5, 8]. Our findings suggested that tumor cells containing WT p53 and the S47 variant might differ in mitochondrial metabolism. To address this issue we assessed oxygen consumption rate and mitochondrial fitness using a Seahorse Bio-Analyzer. For this analysis we used E1A/RAS transformed mouse embryo fibroblast lines from the WT and S47 mouse; all analyses were performed on two independent clones of each genotype that were described previously [7, 9]. This analysis revealed consistent decreases in oxygen consumption rate in S47 transformed cell lines; it also revealed decreased fitness of S47 mitochondria, as assessed by the blunted response to the uncoupling reagent FCCP in S47 tumor cells (Figure 1A, dotted line B). This decrease in oxygen consumption in S47 tumor cells was accompanied by increased extra-cellular acidification rate (ECAR, Figure 1B), which is indicative of increased lactate production and increased aerobic glycolysis. To test the hypothesis that S47 tumor cells show increased aerobic glycolysis, or Warburg metabolism, we performed the glycolytic rate assay using the Seahorse. This analysis confirmed increased glycolysis, at both the basal and metabolically stressed states, in S47 tumor cells compared to WT p53 (Figure 1C–1E).

### Decreased RRAD and increased membrane-localized GLUT1 in S47 tumor cells

**Figure 2:**
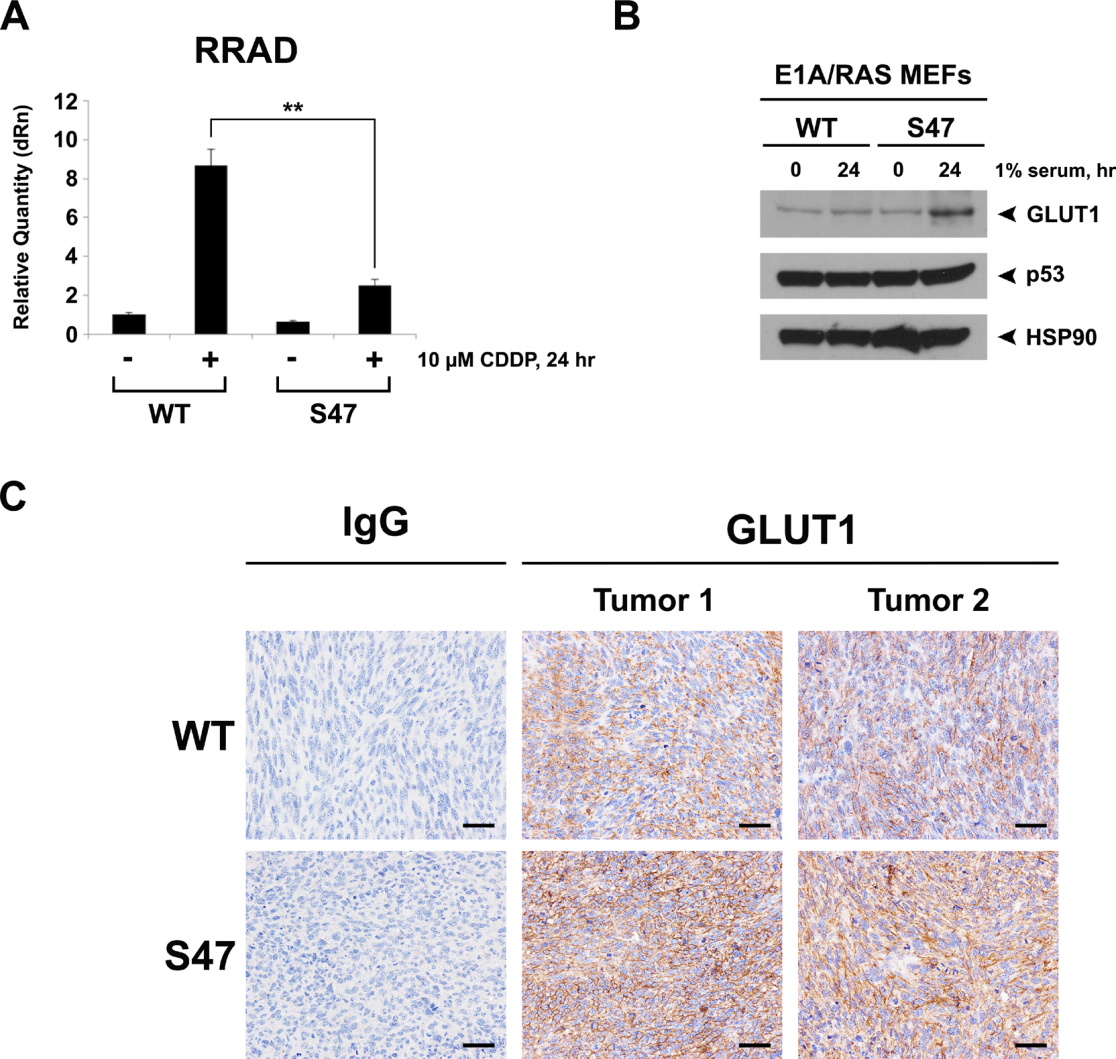
Decaresed induction of RRAD and increased expression of GLUT1 in cells and tumors containing the S47 variant (**A**) WT and S47 E1A/RAS MEFs were treated with 10 μM Cisplatin (CDDP) for 24 hours. Cellular RNA was isolated and used for quantitative reverse transcription-PCR analysis, and levels of RRAD were normalized to cyclophilin A. Values shown reflect the mean ± standard deviation of technical replicates. Shown are representative data of two independent clones of each genotype. (**B**) Western blot analysis of GLUT1, p53 and HSP90 (control) in WT and S47 E1A/RAS MEFs subjected to nutrient-deprived conditions (1% serum) for 24 hours. (**C**) GLUT1 protein staining in formalin fixed paraffin embedded tumors from WT and S47 E1A/RAS cells. Staining using Rabbit IgG was used as a negative control (scale bar, 100 µm). Shown are representative data from 5 independent tumors of each genotype.

We next sought to determine the potential mechanism for increased aerobic glycolysis in S47 tumor cells. Toward this end, we used quantitative RT-PCR to assess the ability of the S47 variant to transactivate the p53 target genes SCO2 and GLS2, which we previously reported are decreased in non-transformed S47 cells compared to WT [5]. We also analyzed transactivation of RRAD, as RRAD is a known p53 target gene whose protein product negatively regulates glycolysis [10, 11]. In transformed WT and S47 E1A/RAS cells, we saw modest differences in the transactivation of GLS2 and SCO2 [9]. However, we saw markedly reduced transactivation of RRAD in S47 transformed cells compared to WT (Figure 2A). We also noted decreased basal levels of RRAD in untreated S47 cells (Figure 2A).

RRAD inhibits glycolysis by negatively regulating the activity of the glucose importer GLUT1 in response to metabolic stress [10, 11]. We found that metabolic stress caused significantly increased GLUT1 levels in S47 tumor cells compared to WT (Figure 2B). Consistent with this, we found that xenograft tumors from S47 E1A/RAS cells showed markedly increased membrane localization of GLUT1, compared to equal size tumors from E1A/RAS-transformed cells with WT p53 (Figure 2C).

### Increased sensitivity of S47 tumor cells to 2-deoxy-glucose

**Figure 3:**
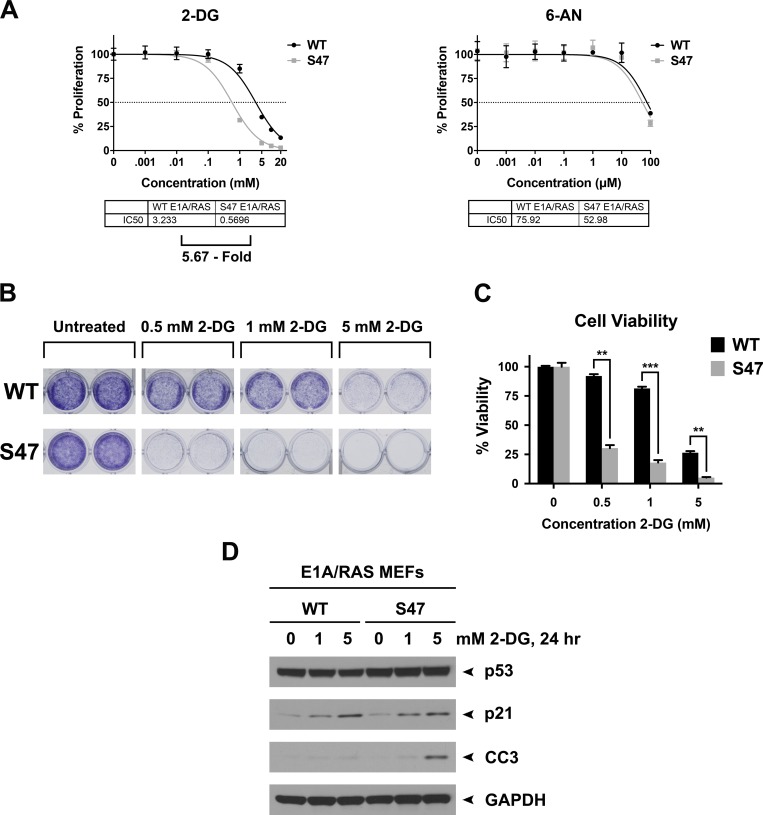
Tumor cells with the S47 variant of p53 show increased sensitivity to 2-Deoxy-D-glucose (2-DG) (**A**) IC_50_ analysis using Alamar Blue assays in WT and S47 E1A/RAS tumor cells treated with 2-DG (left panel) or the pentose phosphate pathway inhibitor 6-aminonicotinamide (6-AN, right panel). (**B**) WT and S47 E1A/RAS MEFs were plated at a density of 10,000 cells per well in 24-well plates. The next day, cells were treated with the indicated concentrations of 2-DG for 48 hours. Cells were then fixed with 10% formalin and stained with 0.5% Crystal Violet. Shown are representative images of duplicate wells from experiments performed in triplicate, which were repeated twice. (**C**) Quantitation of (B) as per absorbance at 590 nm. ^**^p < 0.01, ^***^p < 0.001. (**D**) 700,000 WT and S47 E1A/RAS MEFs were plated in 100-mm dishes. The next day, cell were treated with the indicated doses of 2-DG for 24 hours. Cell lysates were subjected to Western blot analysis, and immunoblotted for p53, cleaved caspase-3 (CC3), p21, and GAPDH (loading control).

The increased glycolysis in S47 tumor cells, compared to those with WT p53, suggested that the former might be more sensitive to glycolysis inhibitors like 2-deoxy-glucose (2-DG). To test this hypothesis, we assessed the IC_50_ of 2-DG in WT and S47 E1A/RAS tumor cells. We also compared the effects of 2-DG on cell survival using crystal violet staining and Western blots for cleaved caspase-3, a marker of apoptosis. We found that S47 tumor cells were 5.7-fold more sensitive to 2-DG (Figure 3A, left panel). In contrast, there was no difference in the sensitivity of these cell lines to the inhibitor of the pentose phosphate pathway, 6-aminonicotinamide (Figure 3A, right panel). Crystal violet staining of treated cells confirmed the markedly increased sensitivity of S47 tumor cells to 2-DG (Figure 3B and 3C) and Western blot analysis indicated that there was increased programmed cell death (cleaved caspase-3) in S47 tumor cells following treatment with 2-DG, compared to WT (Figure 3D). These combined data suggest that the increased glycolysis in S47 tumor cells may constitute a therapeutically targetable vulnerability.

## DISCUSSION

WT p53 is known to suppress glycolysis, and mutations in *TP53* confer increased glycolytic potential and Warburg metabolism to tumor cells [12–14]. This increased aerobic glycolysis, or Warburg metabolism, is believed to enable tumor cells to shuttle glycolytic intermediates into the synthesis of fatty acids and nucleic acids, and to thus enable enhanced generation of biomass. We previously showed that S47 cells transformed with the oncogene combinations E1A/RAS or MYC/RAS show increased transformed properties, compared to transformed cells with WT p53. As one example, S47 tumor cells show markedly increased growth as xenograft tumors, along with increased vascularization; they also show increased proliferation in low-serum conditions [7]. These findings led us to hypothesize that the metabolism of WT and S47 tumor cells might be distinct. We show here-in that transformed S47 tumor cells show increased use of glycolysis for energy. This finding is interesting in light of our previously published reports that the codon 72 variants in p53 also show differences in metabolism [3, 15, 16]. We also find that S47 tumor cells are more sensitive to the glycolytic inhibitor 2-DG. We posit that the increased reliance of S47 tumor cells on glycolytic metabolism represents a new actionable pathway for therapeutic intervention. Interestingly, previous studies have shown that cisplatin and 2-DG can synergize to kill tumor cells by increasing reactive oxygen species [17]; this supports the combination of these two agents in S47 tumors. In addition to our previous efforts [7, 9], the data presented here further emphasize the plausibility of personalizing therapy for African-descent individuals with the S47 variant who have cancer.

## MATERIALS AND METHODS

### Cell culture and reagents

Wild type (WT) and S47 E1A/RAS transformed MEFs (two independent clones of each genotype) were generated previously [7]. E1A/RAS MEFs were grown in DMEM (Corning Cellgro™, Corning, NY, USA) supplemented with 10% Fetal Bovine Serum (HyClone™, GE Healthcare Life Sciences) and 1% penicillin/streptomycin (Corning Cellgro™). Cells were grown in a 5% CO_2_ humidified incubator at 37° C. Cell lines were tested for mycoplasma every six months. Cisplatin was purchased from MedChem Express (HY-17394, Monmouth Junction, NJ, USA) and freshly made as a 5 mM stock in water. 2-Deoxy-D-glucose (2-DG, D6134-1G) and 6-Aminonicotinamide (6-AN, A68203-1G) were purchased from Millipore Sigma (Burlington, MA, USA).

### Antibodies, Western blotting, and immunohistochemistry (IHC)

GLUT1 (12939S, used for Western blotting), HSP90 (4877S), p53 (2524S), Cleaved Caspase-3 (9661S) and GAPDH (2118S) antibodies were purchased from Cell Signaling Technologies (Danvers, MA, USA), and p21 (ab7960) antibody was purchased from Abcam (Cambridge, MA, USA). GLUT1 (ab652) was used for IHC at a concentration of 1:500 and was purchased from Abcam (Cambridge, MA, USA). Normal rabbit IgG was used as a negative control. For Western blotting, 50–100 µg of whole cell lysate was resolved over SDS PAGE gels using pre-cast 10% NuPAGE Bis-Tris gels (Thermo Fisher Scientific) and transferred onto PVDF membranes (IPVH00010, pore size: 0.45 µm) (Millipore Sigma) prior to analysis. Horseradish peroxidase-conjugated secondary antibodies were purchased from Jackson ImmunoResearch (West Grove, PA, USA). IHC analysis was performed as described [7].

### Cell viability assays

For Alamar Blue cell viability assays, E1A/RAS MEFs were plated at a density of 2,000 cells per well on a 96-well plate and left to grow overnight in complete growth medium at 37° C. Cells were then treated with the indicated compounds at various concentrations and incubated for 48 hours, followed by incubation with Alamar Blue (DAL1025) (Thermo Fisher Scientific) for 2 hours at 37° C. Viability was read out according to the manufacturer’s protocol using a Synergy™ HT plate reader (BioTek, Winooski, VT, USA). For Crystal Violet staining, cells were plated at a density of 10,000 cells per well on a 24-well plate and subjected to the indicated concentrations of 2-DG. After 48 hours, cells were washed with PBS, fixed with 10% Neutral Buffered Formalin for 15 minutes, stained with 0.5% Crystal Violet (C3386, Sigma-Aldrich) for 1 hour, rinsed with deionized water, and air dried overnight. The next day, the crystal violet solution was solubilized using 10% acetic acid for 20 minutes and absorbance (590 nm) was read using a PerkinElmer EnVision 2104 Multilabel Reader (Waltham, MA, USA). Data analysis, including logarithmic transformation, graphical analysis, and IC_50_ calculations, were conducted using GraphPad Prism 7.0 (GraphPad Software Inc., La Jolla, CA, USA).

### Mitochondrial oxygen consumption rates (OCR)

The XF Cell Mito Stress Test was performed as previously described [18]. Briefly, forty thousand E1A/RAS MEFs were plated in Seahorse 96-well cell culture microplates coated with Cell-Tak (Corning^™^) and subjected to the Seahorse XF Cell Mito Stress Test, according to manufacturer’s protocol. Briefly, cell medium was replaced with Seahorse XF Base Medium (supplemented with 100 mM Pyruvate, 200 mM Glutamine, and 2.5 M Glucose) and incubated in a non-CO_2_ incubator for one hour before the start of the assay. Basal OCR was measured using the Seahorse XFe96 Extracellular Flux analyzer. Measurements were performed after injection of three compounds affecting bioenergetics: 1 μM oligomycin (Seahorse Bioscience, North Billerica, MA, USA), 1 μM carbonyl cyanide 4-(trifluoromethoxy)phenylhydrazone (FCCP) (Seahorse Bioscience) and 0.5 μM Rotenone/Antimycin A (Seahorse Bioscience). Data are representative of three biological replicates.

### Glycolytic rate assays

Sixty thousand E1A/RAS MEFs were plated in Seahorse 96-well cell culture microplates coated with Cell-Tak (Corning^™^) and subjected to the Seahorse XF Glycolytic Rate Assay, according to manufacturer’s protocol. Media was changed to Agilent Seahorse XF Base Medium without Phenol Red supplemented with 5.0 mM HEPES prior to the start of the assay. Measurements were performed after injection of three compounds affecting bioenergetics: 0.5 μM Rotenone/Antimycin A (Seahorse Bioscience), and 50 mM 2-deoxy-D-glucose (2-DG) (Seahorse Bioscience). Data are representative of three biological replicates.

### qRT-PCR

E1A/RAS MEFs were lysed using QIAshredder columns (Qiagen). Total RNA was isolated and analyzed as previously described [5]. Equal amounts of RNA from all conditions were used to create cDNA using a high-capacity reverse transcription kit (Applied Biosciences, 4368814). qPCR for RRAD was performed using primers 5′AGACGGACCTGAAGCAGAA3′ and 5′TTTCTCAAAGCTGCCCTTGT3′. Data analysis of fold changes in gene transcription was performed using the MxPro program (Stratagene). RNA expression levels were normalized to the housekeeping gene cyclophilin A.

### Statistical analysis of data

All data are reported as the mean ± standard deviation. The two-tailed unpaired Student *t*-test was performed using GraphPad Prism 7.0 software. *p* values are as indicated: ^*^= *p* < 0.05, ^**^= *p* < 0.01, ^***^= *p* < 0.001, and n.s. = *p* > 0.05.
